# Structure and Phylogenetic Relationships of Scolopacidae Mitogenomes (Charadriiformes: Scolopacidae)

**DOI:** 10.3390/cimb46060369

**Published:** 2024-06-19

**Authors:** Quanheng Li, Peiyue Jiang, Mingxuan Li, Jingjing Du, Jianxiang Sun, Nuo Chen, Yu Wu, Qing Chang, Chaochao Hu

**Affiliations:** 1 School of Life Sciences, Nanjing Normal University, Nanjing 210023, China; lqh48325@outlook.com (Q.L.); 18652961559@163.com (M.L.); 15533917216@163.com (J.D.); 15951168432@163.com (J.S.); 18652939025@163.com (N.C.); 15161817540@163.com (Y.W.); 2 Jiangsu Key Laboratory for Biodiversity and Biotechnology, School of Life Sciences, Nanjing Normal University, Nanjing 210023, China; jpy100304@163.com; 3 Analytical and Testing Center, Nanjing Normal University, Nanjing 210046, China

**Keywords:** Scolopacidae, phylogeny, mitochondrial genome, genomics

## Abstract

The family Scolopacidae presents a valuable subject for evolutionary research; however, molecular studies of Scolopacidae are still relatively understudied, and the phylogenetic relationships of certain species remain unclear. In this study, we sequenced and obtained complete mitochondrial DNA (mtDNA) from *Actitis hypoleucos* and partial mtDNA from *Numenius arquata*, *Limosa limosa,* and *Limnodromus semipalmatus*. The complete mtDNA contained 13 protein-coding genes (PCGs), two ribosomal RNA genes, 22 tRNA genes, and a control region. Scolopacidae contained three types of start codons and five types of stop codons (including one incomplete stop codon, T--). In 13 protein-coding genes, average uncorrected pairwise distances (Aupd) revealed that *ATP8* was the least conserved while *COX3* had the lowest evolutionary rate. The ratio of Ka/Ks suggested that all PCGs were under purifying selection. Using two methods (maximum likelihood and Bayesian inference) to analyze the phylogenetic relationships of the family Scolopacidae, it was found that the genera *Xenus* and *Actitis* were clustered into another sister group, while the genus *Phalaropus* is more closely related to the genus *Tringa*. The genera *Limnodromus*, *Gallinago,* and *Scolopax* form a monophyletic group. This study improves our understanding of the evolutionary patterns and phylogenetic relationships of the family Scolopacidae.

## 1. Introduction

The order Charadriiformes is a complex group with 390 extant species [1,2]. It has a wide distribution and occupies diverse habitats. The phylogenetic relationships within Charadriiformes based on morphological characteristics and biochemical methods show distinct differences [3,4]. Molecular studies have classified Charadriiformes into the three monophyletic suborders Scolopaci, Lari, and Charadrii [5,6]. The family Scolopacidae, which includes a diverse array of bird species with about 97 species in 15 genera [7], is known for its strong migratory capabilities. These birds breed in the Arctic and sub-Arctic regions and winter in tropical areas, making them a representative group for bird migration studies. Mitochondrial genomes (mitogenomes), characterized by their small size, maternal inheritance, high mutation rate, low occurrence of recombination, and fast evolution, have become one of the most commonly used molecular markers in evolutionary studies. They are widely used in molecular systematics, phylogenetics, conservation genetics, and evolutionary analysis [8,9]. Animal mitogenomes are generally circular molecules ranging from 15–20 kb in size [10], containing 22 tRNA genes, 13 protein-coding genes (PCGs), two rRNA genes (12S RNA and 16S rRNA), and one control region (D-loop) that includes signals necessary for replication and transcription [11,12]. Phylogenetic analyses based on the *Cyt b* and the whole mtDNA sequences revealed that the slender-billed curlew (*Numenius tenuirostris*) was closely related to the Eurasian curlew (*N. arquata*), the Eastern curlew (*N. madagascariensis*)*,* and long-billed curlew (*N. americanus*) [13]. Lopez et al., using newly sequenced mitogenomes, clarified the taxonomic status of the Tahiti sandpiper (*Prosobonia leucoptera*) and Tuamotu sandpiper (*P. parvirostris*) within the family Scolopacidae and relationships with turnstones and calidrine sandpipers, providing insights into the evolutionary mechanisms of sedentary populations within highly migratory Scolopacidae [14].

At present, the main threats to Scolopacidae include habitat loss in breeding, migration, and wintering areas, while increased hunting pressure and adverse climate factors exacerbated their survival crisis, attracting the attention of researchers [15,16]. Many species in the family Scolopacidae were capable of long-distance migration, and inevitably faced challenges in energy synthesis, storage, and metabolic efficiency during migration. However, the current research on Scolopacidae still fails to provide a clear explanation for their evolutionary mechanisms. Molecular-level studies of Scolopacidae are not only helpful in clarifying their phylogenetic relationships but also offer new insights into the evolution of Scolopacidae [17,18].

Currently, compared to other biological groups, basic genomic data on Scolopacidae are still relatively scarce, limiting the study of their taxonomic status and evolution. Common sandpiper (*Actitis hypoleucos*), Asian dowitcher (*Limnodromus semipalmatus*), black-tailed godwit (*Limosa limosa*), and *N. arquata* are representative species of Scolopacidae, and their tissue samples are well stocked in laboratories. Considering the lack of basic molecular research on Scolopacidae, this study was conducted to sequence and analyze the whole mitogenomes of the four species mentioned above, to clarify the phylogeny and evolution of the family Scolopacidae. The results may help to clarify the phylogenetic relationships of the family Scolopacidae and provide useful information and data for understanding evolutionary and taxonomic research on Scolopacidae.

## 2. Materials and Methods

### 2.1. Sampling and DNA Extraction

After being identified using morphological methods, feathers and muscle tissues of *N. arquata*, *A. hypoleucos*, *L. limosa*, and *L. semipalmatus* collected in the eastern coastal areas of China were preserved in absolute ethanol at −20 °C under the voucher numbers of NJNU-Narq02, NJNU-Ahyp07, NJNU-Llim08, and NJNU-Lsem09, respectively. The species morphological identification was according to A Checklist on the Classification and Distribution of the Birds of China (Third Edition) [19]. Feathers and muscle tissues were preserved in absolute ethanol at −20 °C under the voucher numbers of NJNU-Narq02, NJNU-Ahyp07, NJNU-Llim08, and NJNU-Lsem09, respectively. DNA was extracted from the samples using phenol–chloroform [20] and ethanol, and the concentration of DNA was determined by a Nanodrop 1000 spectrophotometer (Thermo Scientific, Waltham, MA, USA) to ensure DNA purity with an A260/A280 ratio between 1.8 and 2.0. Subsequently, DNA quality was analyzed using agarose gel electrophoresis. An amount of 5 µL of the DNA sample was electrophoresed in a 1% agarose gel at 130 V for 30 min to check for contamination or degradation. Qualified DNA samples were then diluted and stored at −20 °C for further experiments.

### 2.2. PCR Amplification and Mitochondrial Genome Sequencing

We used 13 pairs of PCR primers obtained from previous studies to amplify the mitogenomes [21]. PCR amplifications were carried out in a final reaction volume of 30 µL, which contained 15 µL of 2× Rapid Taq Master Mix, 0.7 µL of each forward and reverse primer, 12.6 µL of sterile distilled water (ddH_2_O), and 1 µL of template DNA. The PCR cycling conditions with universal primers consisted of 35 cycles, each comprising an initial denaturation at 95 °C for 5 min, denaturation at 95 °C for 30 s, annealing at 55 °C for 30 s, and extension at 72 °C for 90 s. The PCR products were assessed through electrophoresis in a 1.00% agarose gel, and were sequenced directly by Beijing Tsingke Biotech Co., Ltd. (Beijing, China).

### 2.3. Mitogenome Annotation and Sequence Analysis

The SeqMan program (DNAstar, Madison, WI, USA) was used for splicing, assembly, and proof-reading. The circular map was generated using CGView (https://proksee.ca/, accessed on 13 April 2024) [22], ORF Finder was used to identify 13 PCGs, and MEGA11 was used to compare the mitochondrial sequences. tRNAscan-SE2.0 [23] (https://github.com/tRNAscan-SE/tRNAscan-SE, accessed on 13 April 2024) and ARWEN version 1.2 [24] (http://130.235.244.92/ARWEN/, accessed on 13 April 2024) were used for predicting and illustrating all tRNAs’ secondary structures encoded in the mitogenomes. Publicly available mitogenome data of Scolopacidae were retrieved from the GenBank database. Although there are many misidentifications in data from GenBank, it is the most accurate database available to us. MEGA11 was used to analyze the nucleotide composition and codon usage of the mitogenomes of 40 nucleotide sequences from 33 species of Scolopacidae [25], including the four species mentioned above. The mitogenomes’ strand asymmetry was calculated by the following formulas: AT skew = (A − T)/(A + T) and GC skew = (G − C)/(G + C) [26]. MEGA11 was also used to calculate the number of variable sites (Vs), parsimony informative sites (Pis), singletons (S), average uncorrected pairwise distances (Aupds), and the estimated transition/transversion bias (ts/tv) for the 33 species of Scolopacidae and a total of 40 protein-coding sequences [27]. Non-synonymous substitution rates (Ka, π modified) and synonymous substitution rates (Ks, π modified) for each protein-coding gene were calculated in DnaSP V6 for selective pressure analysis [28].

### 2.4. Phylogenetic Analysis

The 40 nucleotide sequences of 13 PCGs, 12S rRNA and 16S rRNA genes, from 33 species of Scolopacidae (Table 1), were aligned using MAFFT version 7.313 [29] implemented in PhyloSuite version 1.2.3 with 13,600 bp in length [30,31]. ModelFinder was used to select the best partitioning strategy and evolutionary model for the gene dataset, with model fit assessed using the Bayesian information criterion (BIC). Phylogenetic trees were constructed using the maximum likelihood (ML) and Bayesian inference (BI) methods, with ML analysis performed using IQ-TREE version 1.6.8 [32] and BI analysis using MrBayes version 3.2.6 [33]. For ML analysis, node support values were evaluated using 1000-bootstrap resampling with the optimal partition model (GTR+F+I+I+R4). For BI analysis, two simultaneous runs (4 chains) were conducted for 50 million generations, sampling every 1000 generations, with each data partition applying an independent model. When the average standard deviation of split frequencies (ASDSF) is below 0.01, it indicates that the runs have converged. All phylogenetic trees were visualized using iTOL v6.8.2 [34].

## 3. Results and Discussion

### 3.1. Structure of Mitogenome

**Figure 1:**
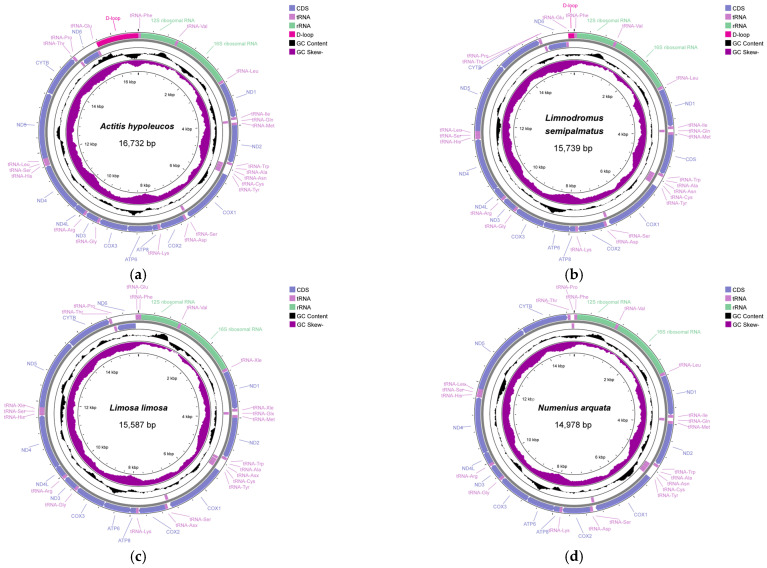
Gene maps of the mitogenomes of (**a**) *A. hypoleucos*, (**b**) *L. semipalmatus*, (**c**) *L. limosa*, and (**d**) *N. arquata*. Arrows indicate the orientation of gene transcription. PCGs are shown as blue-purple arrows, rRNA genes as green arrows, tRNA genes as pink arrows, and the control region as dark pink arrows. Ticks in the inner cycle indicate the sequence length. The black ring indicates the GC content (outward and inward peaks showing above- or below-average GC content, respectively). The purple ring indicates the GC skew.

We sequenced and annotated the mitogenomes of *A. hypoleucos* (PP727181), *L. semipalmatus* (PP737170), *N. arquata* (PP737172), and *L. limosa* (PP737171) and obtained one complete (*A. hypoleucos*) and three nearly complete mitogenomes (*L. semipalmatus*, *N. arquata*, and *L. limosa*) (Figure 1 and Tables S1–S4). The gene arrangement pattern of vertebrate mitogenomes is highly conserved, and the four species conform to this arrangement [9]. The complete mitogenome of *A. hypoleucos* is 16,732 bp in length, containing 13 PCGs, 12S rRNA, 16S rRNA, and 22 tRNA genes, and one D-loop. Compared to the complete mitogenomes, the sequencing results of *L. semipalmatus* and *L. limosa* are missing parts of the D-loop, while *N. arquata* is missing one PCG (*ND6*), one tRNA gene (tRNA^Glu^), and the D-loop.

Twenty-four intergenic spacers or overlaps were found in the mitogenome of *A. hypoleucos*, which ranged from 1 to 15 bp, and the longest spacer was located between tRNA^Leu(UUR)^ and *ND1*. The longest overlap was located between *COX1* and tRNA^Ser(UCN)^. We found 25 intergenic spacers or overlaps in the partial mitogenome of *L. semipalmatus*, and the longest spacer was located between tRNA^Pro^ and *ND6*, with the longest overlap occurring between *ATP8* and *ATP6*. The partial mitogenome of *L. limosa* contains 23 gene spacers and overlaps; the longest gene overlap, identical to that in *A. hypoleucos*, is between *COX1* and tRNA^Ser(UCN)^; the longest spacer, the same as in *L. semipalmatus*, is between tRNA^Pro^ and *ND6*. These features had also been observed in other species of Charadriiformes and Passeriformes [15,35,36,37,38]. Furthermore, a 10 bp gene overlap between *ATP8* and *ATP6* and a 7 bp overlap between *ND4* and *ND4L* were observed in these three species, consistent with observations in most vertebrate mitogenomes [39]. An extra nucleotide (C: cytosine) was identified at position 174 in the *ND3* gene of *L. semipalmatus*, *L. limosa*, and *A. hypoleucos*. In fact, this untranslated “C” also appeared in other species such as *C. pygmeus*, *Charadrius dubius*, and *Elanus caeruleus*, as well as some sea turtles [40,41,42,43].

The base composition and the values of the AT and GC skews for 30 species of Scolopacidae were calculated and are shown in Table S5. The complete mitogenome of *A. hypoleucos* consists of 25.99% T, 29.21% C, 31.37% A, and 13.43% G, which showed a higher A+T content (57.36%) than the average A+T content (56.71%) of Scolopacidae. The A+T content of Scolopacidae varied, ranging from 55.34% (*A. interpres*) to 58.70% (*P. parvirostris*). A positive value of AT skew (0.09) and a negative value of GC skew (−0.37) was observed in *A. hypoleucos*, a characteristic that is common among all Scolopacidae species (Table S5). The positive AT skew values ranged from 0.09 to 0.13 and the negative GC skew values ranged from −0.39 to −0.35, which suggested more A, C than T, G in the mitogenomes of Scolopacidae.

### 3.2. Composition and Mutations of Protein-Coding Genes

The total length of the PCGs in *A. hypoleucos* was 11,394 bp, accounting for 68.10% of the entire length, with the sizes of PCGs ranging from 168 bp (*ATP8*) to 1815 bp (*ND5*). The total lengths of the 13 PCGs for *L. semipalmatus* and *L. limosa* were the same as that of *A. hypoleucos*. All PCGs were located on the H-strand except for *ND6*. Most PCGs showed a positive AT skew value and a negative GC skew value (Table S6). *ND1* showed slight negative AT skew values in *A. hypoleucos*, *L. semipalmatus*, *N. arquata*, and *L. limosa* (−0.05, −0.02, −0.03, and −0.02, respectively), and significant negative AT skews were observed in *ND6* of *A. hypoleucos* and *L. semipalmatus* (both −0.54) and of *L. limosa* (−0.50). Moreover, *COX1* of *A. hypoleucos* and *ND4L* of *N. arquata* also showed slight negative AT skew values of −0.03 and −0.01, respectively. Additionally, *ND6* in *A. hypoleucos*, *L. semipalmatus*, and *L. limosa* presented significant positive GC skew values (0.58, 0.62, and 0.53 respectively).

A comparison of each PCG revealed the variation in the family Scolopacidae (Table 2). *ATP8* showed the highest proportion of variable sites (52.12%), parsimony informative sites (44.85%), and singleton sites (7.27%) among PCGs, while *COX1* had the lowest proportion of variable sites (35.59%) and singleton sites (3.94%), and *COX3* had the lowest parsimony informative sites (31.16%). The average uncorrected pairwise distance ranged from 0.11 (*COX3*) to 0.16 (*ATP8*), suggesting that *ATP8* is the least conserved and *COX3* the most conserved. Single base substitution is mainly divided into two categories: transition (ts) and transversion (tv), and tv is more likely to change the sequence of amino acids within a protein than ts [44]. The ts/tv ratios of PCGs reveal the evolutionary patterns of different genes, and, therefore, are widely used in the analysis of selection pressures at the DNA level [45]. Analysis revealed that *ND1* exhibited the highest ts/tv ratio (4.43), and that *Cyt b* exhibited the lowest ts/tv ratio (2.31).

To better understand the evolutionary patterns of 13 PCGs and further investigate the role of selection on Scolopacidae, Ks, Ka, and Ka/Ks were calculated for each PCG, respectively (*ND5* of *T. semipalmata* and *T. guttifer* were deleted from the calculation of Ks, Ka, and Ka/Ks). The values of Ks ranged from 0.564 (*ND3*) to 0.800 (*ATP8*) and the values of Ka ranged from 0.010 (*COX2*) to 0.080 (*ND6*). The highest and the lowest ratios of Ka/Ks appeared in *ND6* (0.127) and *COX1* (0.007), respectively. The ratios of Ka/Ks of 13 PCGs were less than 1, indicating that their evolution was all subjected to varying degrees of purification selection.

### 3.3. rRNA and tRNA Analysis

The length of 12S rRNA for *A. hypoleucos*, *L. semipalmatus*, *N. arquata*, and *L. limosa* was 973 bp, while the lengths of 16S rRNA were 1590, 1598, 1593, and 1592 bp, respectively. The two rRNAs and 14 tRNAs were identified on the H-strand. The 12S rRNA was located between tRNA^Phe^ and tRNA^Val^, and the 16S rRNA was located between tRNA^Val^ and tRNA^Leu(UUR)^, which were separated by tRNA^Val^. In *A. hypoleucos*, the length of the two rRNAs (2563 bp) accounted for 15.32% of the total length. In addition, the A+T content of the rRNA genes accounted for 57.00%, with an AT skew value of 0.18 and a GC skew value of −0.14.

The 22 tRNAs lengths of the four species varied from 64–80 bp, with tRNA^Ser(UCN)^ encoding the longest tRNA and tRNA^Ser(AGN)^ encoding the shortest tRNA. For *A. hypoleucos*, the total length of tRNAs was 1559 bp, which accounted for 9.32% of the mitogenome length, whereas the total lengths of tRNAs for *L. semipalmatus* and *L. limosa* were 1554 and 1562 bp, respectively. High A+T content was observed in tRNAs of *A. hypoleucos*, *L. semipalmatus*, and *L. limosa*, reaching 58.37%, 58.49%, and 57.81%, respectively. All tRNA secondary structures showed a typical cloverleaf structure, except for tRNA^Ser(AGN)^, which did not contain the dihydrouridine arm (Figures S1–S4).

### 3.4. The Usage of Start and Stop Codon

**Figure 2:**
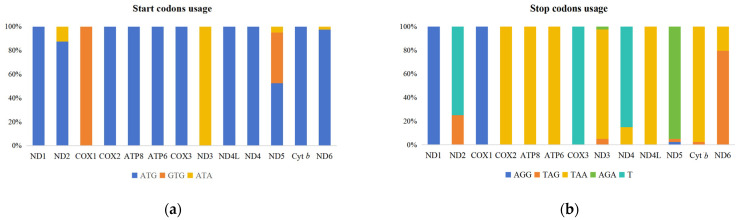
The usage of start (**a**) codons and stop (**b**) codons in the 13 mitochondrial protein-coding genes of family Scolopacidae. All genes are shown in the order of occurrence in the mitogenome starting from *ND1*.

The start and stop codon usage of the 13 PCGs (excluding *ND6* of *N. arquata*) in the mitogenomes of 40 sequences from 33 species of Scolopacidae is shown in Figure 2. Three start codons (ATG, GTG, and ATA) and five stop codons (AGG, TAG, TAA, AGA, and T--) were observed in 13 PCGs. ATG was the most common start codon, accounting for 79.80% of the total start codons in 13 PCGs, followed by GTG (10.96%) and ATA (9.24%). Eight PCGs (*ATP6*, *ATP8*, *COX2*, *COX3*, *Cyt b*, *ND1*, *ND4*, and *ND4L*) use only ATG as the start codon. *COX1* used GTG as the start codon. Of *ND2*, 87.50% used ATG as the start codon, and the rest were ATA, including *S. rusticola* (KM434134), *G. gallinago* (MZ157405 and MW865755), and *G. stenura* (KY056596 and KY888681). Only *T. guttifer* (MK905885) uses ATA as the start codon for *ND6*, while the other species use ATG as the start codon. *ND5* contained three types of start codons, ATG (52.50%), GTG (42.50%), and ATA (5.00%).

PCGs ended with either a complete stop codon (TAA, TAG, AGG, AGA) or an incomplete stop codon (T--), which are thought to be completed by posttranscriptional modifications like polyadenylation [45]. Four PCGs (*ATP6*, *ATP8*, *COX2*, and *ND4L*) used TAA as the stop codon, two PCGs (*COX1* and *ND1*) used AGG as the stop codon, and one PCG (*COX3*) used T-- as the stop codon. TAA was the most common stop codon, accounting for 48.12% of the total stop codons, followed by T-- (20.38%). The stop codon T-- was also detected in *ND2* and *ND4*, accounting for 75.00% and 85.00%, respectively, and this phenomenon also occurred in other species [46]. The remaining 25.00% of *ND2* used TAG as the stop codon, including *L. semipalmatus*, *L. limosa*, and *A. hypoleucos*, while 15.00% of *ND4* used TAA as the stop codon. Almost all *Cyt b* used TAA as the stop codon, but it is worth mentioning that only *S. rusticola* used TAG as the stop codon. In *ND3*, TAA was used as the stop codon in all species except *P. parvirostris* and *N. madagascariensis* (KY230384 and MW930394), which used AGA and TAG as the stop codons, respectively. *ND5* mainly used AGA as the stop codon, and only *G. stenura* (KY888681) and *A. hypoleucos* used TAG and AGG as the stop codons, respectively. *ND6* in most species used TAG as the stop codon, while a few used TAA as the stop codon.

### 3.5. Analysis of Codon Usage for A. hypoleucos, L. semipalmatus, L. limosa, and N. arquata

**Figure 3:**
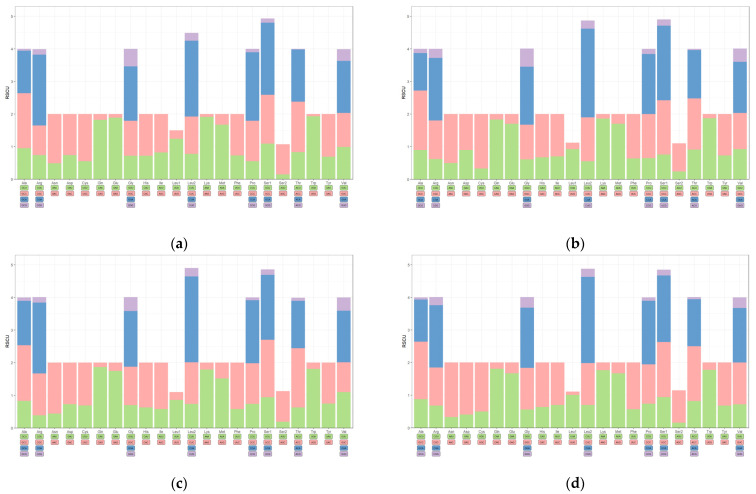
The relative synonymous codon usage (RSCU) of the 13 PCGs of (**a**) *A. hypoleucos*, (**b**) *L. semipalmatus*, (**c**) *L. limosa*, and (**d**) *N. arquata;* the stop codons were not included.

The relative synonymous codon usage (RSCU) values for the 13 PCGs of four species (*A. hypoleucos*, *L. semipalmatus*, *L. limosa,* and *N. arquata*) is summarized in Figure 3, and their base composition is calculated in Table 3. The PCGs of three species consisted of 3787 codons, excluding the start and stop codons. The most frequently used amino acid in *A. hypoleucos*, *L. semipalmatus*, *L. limosa,* and *N. arquata* was leucine (Leu), with 17.59%, 17.51%, 17.45%, and 16.90%, respectively, followed by threonine (Thr), with 9.40%, 9.51%, 9.16%, and 9.45%, while the least frequently used was cysteine (Cys) with 0.77%, 0.79% 0.77%, and 0.63%, respectively. All PCGs of the three species showed a slight positive value of AT skew (0.03, 0.04, 0.04, and 0.06 for *A. hypoleucos, L. semipalmatus*, *L. limosa,* and *N. arquata,* respectively) and a larger negative value of GC skew (−0.39, −0.40, −0.39, and −0.43 for *A. hypoleucos*, *L. semipalmatus*, *L. limosa,* and *N. arquata,* respectively). The skew rate at different codon positions showed some regularity. All of the second-codon positions showed a negative value of the AT skew. Additionally, the positive values of the AT skew and negative values of the GC skew at the third-codon positions (wobble positions) were particularly prominent, with the highest positive values of the AT skew being 0.38 (*L. semipalmatus* and *L. limosa*) and the lowest negative value of GC skew being −0.77 (*A. hypoleucos*), except for *N. arquata*.

### 3.6. Phylogenetic Analysis

**Figure 4:**
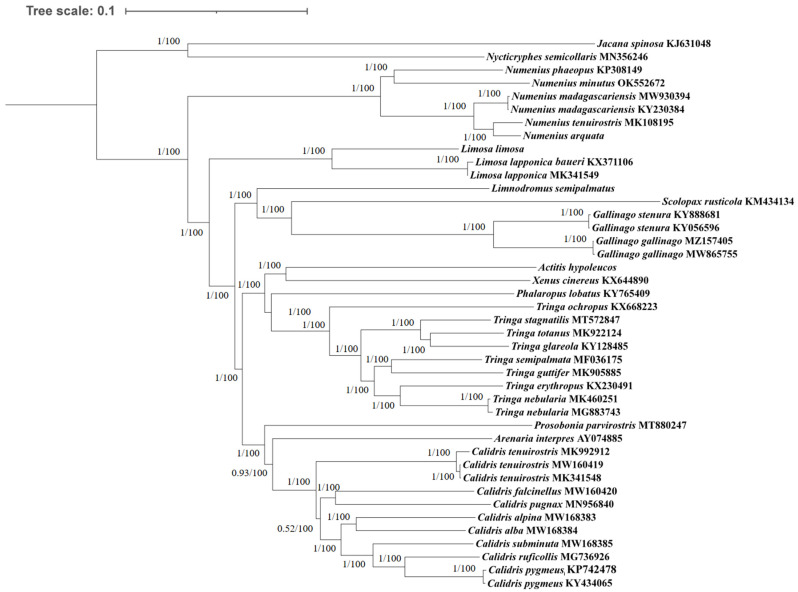
The phylogenetic trees of family Scolopacidae constructed with mitochondrial genes (concatenated 13 protein-coding genes, 12S and 16S rRNA), using Bayesian inference (BI) and maximum likelihood (ML). Numbers at nodes represent the Bayesian posterior probabilities and maximum likelihood bootstrap values, separated by “/”.

Phylogenetic analysis with two inference methods (ML and BI) of 13 mitochondrial protein-coding genes (excluding *ND6* of *N. arquata*), the 12S gene, and the 16S gene for 33 species of Scolopacidae revealed identical topologies, which were highly supported by bootstrap and posterior probabilities at most nodes, and in the analyses, *N. semicollaris* and *J. spinosa* were considered as outgroups (Figure 4). Based on the currently known mitogenomes of the family Scolopacida, we classified the family into the genera *Numenius*, *Limosa*, *Limnodromus*, *Scolopax*, *Gallinago*, *Actitis*, *Xenus*, *Phalaropus*, *Tringa*, *Prosobonia*, *Arenaria,* and *Calidris*, which was in agreement with previous research [1]. The phylogenetic tree results showed that *N. arquata* and *N. tenuirostris* were closely related, and the group of *N. arquata, N. tenuirostris* plus *N. madagascariensis* was sister to the *N. minutus* plus *N. phaeopus* group. Genus *Phalaropus* was found to be more closely related to genus *Tringa,* and genera *Xenus* and *Actitis* were clustered into another sister group, contrary to previous findings suggesting that *Xenus* was closer to *Tringa* [1,15]. The genera *Limnodromus*, *Gallinago,* and *Scolopax* formed a monophyletic group. Our result was also in congruence with previous findings that genus *Calidris* is not monophyletic [47]. In addition, another phylogenetic tree for 33 Scolopacidae species was constructed by using two methods (ML and BI), based on 12 PCGs (excluding *ND6* in family Scolopacidae), the 12S gene, and the 16S gene, with *N. semicollaris* and *J spinosa* as outgroups, which had the same results as the previous ones (Figure S5).

## 4. Conclusions

We sequenced and annotated the mitogenome of four species of Scolopacidae (*A. hypoleucos*, *L. semipalmatus*, *L. limosa*, and *N. arquata*). An extra non-coding “C” was found at position 174 in the *ND3* of *L. semipalmatus, L. limosa,* and *A. hypoleucos*. The mitogenomes of the Scolopacidae family exhibited a bias toward A over T, resulting in a positive AT skew, and a preference for C over G, leading to a negative GC skew. The average uncorrected pairwise distances revealed that out of the 13 PCGs, *ATP8* demonstrates the lowest level of conservation, whereas *COX3* exhibits the least rapid evolutionary rate. Analysis of the mitogenome start and stop codons showed that ATG was the most common start codon, while TAA was the most common stop codon. The third-codon position contributes the most to the overall GC bias of the PCGs. Based on 13 PCGs and two ribosomal RNAs, phylogenetic analysis has categorized the family Scolopacidae into 12 genera according to currently available mitogenome data. Our findings suggest that the genus *Phalaropus* is closer to *Tringa*, contrary to previous studies. Additionally, we observed that genera *Gallinago* and *Scolopax* clustered into one group, which was sister to genus *Limnodromus*.

## Figures and Tables

**Table 1:** All complete mitogenomes of family Scolopacidae deposited in GenBank. Bold represents that the mitogenome was sequenced in this study.

Family	Common Name	Scientific Name	Length (bp)	Accession
**Scolopacidae**	**Common sandpiper**	***Actitis hypoleucos***	**16,732**	**PP727181**
	Ruddy turnstone	*Arenaria interpres*	16,725	AY074885
	Sanderling	*Calidris alba*	16,642	MW168384
	Dunlin	*Calidris alpina*	16,791	MW168383
	Ruff	*Calidris pugnax*	16,902	MN956840
	Spoon-billed sandpiper	*Calidris pygmeus*	16,709	KY434065
		*Calidris pygmeus*	16,707	KP742478
	Red-necked stint	*Calidris ruficollis*	16,860	MG736926
	Long-toed stint	*Calidris subminuta*	16,765	MW168385
	Great knot	*Calidris tenuirostris*	16,775	MK992912
		*Calidris tenuirostris*	16,732	MK341548
		*Calidris tenuirostris*	16,678	MW160419
	Broad-billed sandpiper	*Calidris falcinellus*	15,555	MW160420
	Common snipe	*Gallinago gallinago*	16,919	MZ157405
		*Gallinago gallinago*	16,814	MW865755
	Pintail snipe	*Gallinago stenura*	16,899	KY056596
		*Gallinago stenura*	18,153	KY888681
	Bar-tailed godwit	*Limosa lapponica*	16,773	MK341549
		*Limosa lapponica baueri*	16,732	KX371106
	**Black-tailed godwit**	***Limosa limosa***	**15,587**	**PP737171**
	**Asian dowitcher**	***Limnodromus semipalmatus***	**15,739**	**PP737170**
	**Eurasian curlew**	***Numenius arquata***	**14,978**	**PP737172**
	Eastern curlew	*Numenius madagascariensis*	17,668	KY230384
		*Numenius madagascariensis*	17,117	MW930394
	Little curlew	*Numenius minutus*	17,047	OK552672
	Whimbrel	*Numenius phaeopus*	17,091	KP308149
	Slender-billed curlew	*Numenius tenuirostris*	16,705	MK108195
	Red-necked phalarope	*Phalaropus lobatus*	16,714	KY765409
	Tuamotu sandpiper	*Prosobonia parvirostris*	15,590	MT880247
	Eurasian woodcock	*Scolopax rusticola*	16,984	KM434134
	Spotted redshank	*Tringa erythropus*	16,683	KX230491
	Wood sandpiper	*Tringa glareola*	16,804	KY128485
	Nordmann’s greenshank	*Tringa guttifer*	16,835	MK905885
	Common greenshank	*Tringa nebularia*	16,682	MK460251
		*Tringa nebularia*	16,689	MG883743
	Green sandpiper	*Tringa ochropus*	16,906	KX668223
	Willet	*Tringa semipalmata*	16,603	MF036175
	Marsh sandpiper	*Tringa stagnatilis*	16,799	MT572847
	Common redshank	*Tringa totanus*	16,818	MK922124
	Terek sandpiper	*Xenus cinereus*	16,817	KX644890
Jacanidae	Northern jacana	*Jacana spinosa*	17,079	KJ631048
Rostratulidae	South American painted-snipe	*Nycticryphes semicollaris*	18,584	MN356246

**Table 2:** Variation and evolution analysis of 13 PCGs in Scolopacidae.

Gene	%Vs	%Pis	%S	%Aupd	ts/tv	Ks	Ka	Ka/Ks
*ND1*	42.26%	37.54%	4.72%	0.14	4.43	0.793	0.020	0.025
*ND2*	48.51%	42.16%	6.35%	0.15	3.28	0.783	0.042	0.054
*COX1*	35.59%	31.65%	3.94%	0.12	3.96	0.762	0.005	0.007
*COX2*	36.56%	31.57%	4.99%	0.12	4.00	0.739	0.010	0.014
*ATP8*	52.12%	44.85%	7.27%	0.16	2.36	0.800	0.063	0.079
*ATP6*	44.93%	37.89%	7.05%	0.14	2.71	0.704	0.020	0.028
*COX3*	36.14%	31.16%	4.98%	0.11	3.49	0.646	0.011	0.017
*ND3*	43.84%	38.97%	4.87%	0.13	4.35	0.564	0.042	0.074
*ND4L*	43.54%	37.07%	6.46%	0.12	4.33	0.647	0.016	0.025
*ND4*	46.41%	39.29%	7.04%	0.14	2.66	0.687	0.033	0.048
*ND5*	45.68%	38.64%	7.04%	0.13	3.36	0.662	0.078	0.118
*Cyt b*	41.49%	34.65%	6.84%	0.13	2.31	0.655	0.021	0.032
*ND6*	50.00%	40.80%	9.00%	0.14	2.40	0.632	0.080	0.127

Vs: variable sites, Pis: parsimony informative sites, S: singleton sites, Aupd: the average uncorrected pairwise distances. ts/tv: the estimated transition/transversion bias.

**Table 3:** Nucleotide composition and skew rate at different sites of codons in four species.

Species	Codon Site	Proportion of Nucleotides (%)					AT Skew	GC Skew
		T	C	A	G	A+T		
*A. hypoleucos*	1st	23.11	26.01	29.05	21.84	52.15	0.11	−0.09
	2nd	40.35	28.76	18.30	12.60	58.65	−0.38	−0.39
	3rd	20.02	34.51	40.93	4.54	60.95	0.34	−0.77
	Total	27.82	29.76	29.43	12.99	57.25	0.03	−0.39
*L. semipalmatus*	1st	21.73	27.36	28.89	22.02	50.62	0.14	−0.11
	2nd	40.08	28.97	18.22	12.73	58.30	−0.38	−0.39
	3rd	18.17	36.52	40.16	5.15	58.33	0.38	−0.75
	Total	26.66	30.95	29.09	13.30	55.75	0.04	−0.40
*L. limosa*	1st	21.86	27.20	28.68	22.26	50.54	0.13	−0.10
	2nd	40.14	28.76	18.27	12.83	58.41	−0.37	−0.38
	3rd	17.72	37.36	39.40	5.52	57.12	0.38	−0.74
	Total	26.57	31.11	28.78	13.54	55.36	0.04	−0.39
*N. arquata*	1st	21.64	27.68	29.75	20.92	51.39	0.16	−0.14
	2nd	39.72	29.59	18.65	12.04	58.37	−0.36	−0.42
	3rd	17.41	37.86	40.13	4.59	57.54	0.39	−0.78
	Total	26.26	31.71	29.51	12.52	55.77	0.06	−0.43

## Data Availability

The genome sequence data that support the findings of this study are openly available in GenBank of NCBI at https://www.ncbi.nlm.nih.gov/ under accession no. PP727181, PP737170, PP737172 and PP737171.

## Supplementary Materials

The following supporting information can be downloaded at https://www.mdpi.com/article/10.3390/cimb46060369/s1, Figure S1: Secondary structures of the 22 tRNA genes of *A*. *hypoleucos*; Figure S2: Secondary structures of the 22 tRNA genes of *L*. *semipalmatus*; Figure S3: Secondary structures of the 21 tRNA genes of *N*. *arquata*; Figure S4: Secondary structures of the 22 tRNA genes of *L*. *limosa*; Figure S5: Phylogenetic relationships among Scolopacidae species based on mitochondrial genes (concatenated 12 protein-coding genes, 12S and 16S rRNA); Table S1: Organization of the complete mitogenome of *A*. *hypoleucos*; Table S2: Organization of the partial mitogenome of *L*. *semipalmatus*; Table S3: Organization of the partial mitogenome of *N*. *arquata*; Table S4: Organization of the partial mitogenome of *L*. *limosa*; Table S5: The base composition and skew rate of the family Scolopacidae; Table S6: The base composition and skew rate of PCGs.
