# Supplementary material for: Structure and Phylogenetic Relationships of Scolopacidae Mitogenomes (Charadriiformes: Scolopacidae)
Source: Curr Issues Mol Biol. 2024 Jun 19;46(6):6186–98. doi: 10.3390/cimb46060369 (PMC11202466; doi:10.3390/cimb46060369)
Supplement: Supplementary file 1 [file cimb-46-00369-s001.zip › cimb-3029309-supplementary.pdf]

Table S1. Organization of the complete mitochondrial genome of *A. hypoleucos*.

Gene	Strand	Position	Overlap/spacer	Size (bp)	Anticodon
*tRNA^Phe^*	H	1-71		71	GAA
s-rRNA	H	72-1044		973	
*tRNA^Val^*	H	1045-1116		72	TAC
l-rRNA	H	1117-2706	1	1590	
*tRNA^Leu(UUR)^*	H	2708-2781	13	74	TAA
ND1	H	2795-3772		978	
*tRNA^Ile^*	H	3773-3842	9	70	GAT
*tRNA^Gln^*	L	3852-3922	-1	71	TTG
*tRNA^Met^*	H	3922-3990		69	CAT
ND2	H	3991-5029		1039	
*tRNA^Trp^*	H	5030-5098	1	69	TCA
*tRNA^Ala^*	L	5100-5168	3	69	TGC
*tRNA^Asn^*	L	5172-5244	2	73	GTT
*tRNA^Cys^*	L	5247-5313	-1	67	GCA
*tRNA^Tyr^*	L	5313-5383	1	71	GTA
COX1	H	5385-6935	-15	1551	
*tRNA^Ser(UCN)^*	L	6921-7000	2	80	TGA
*tRNA^Asp^*	H	7003-7071	1	69	GTC
COX2	H	7073-7756	1	684	
*tRNA^Lys^*	H	7758-7828	1	71	TTT
ATP8	H	7830-7997	-10	168	
ATP6	H	7988-8671	-1	684	
COX3	H	8671-9454		784	
*tRNA^Gly^*	H	9455-9523		69	TCC
ND3	H	9524-9875	4	352	
*tRNA^Arg^*	H	9880-9949		70	TCG
ND4L	H	9950-10,246	-7	297	
ND4	H	10,240-11,617		1378	
*tRNA^His^*	H	11,618-11,686	1	69	GTG
*tRNA^Ser(AGN)^*	H	11,688-11,751		64	GCT
*tRNA^Leu(CUN)^*	H	11,752-11,822		71	TAG
ND5	H	11,823-13,637	12	1815	
CYTB	H	13,650-14,792	2	1143	
*tRNA^Thr^*	H	14,795-14,870	7	76	TGT
*tRNA^Pro^*	L	14,878-14,947	12	70	TGG
ND6	L	14,960-15,481	3	522	
*tRNA^Glu^*	L	15,485-15,558		74	TTC
D-loop	H	15,559-16,732		1174	

Table S2. Organization of the partial mitochondrial genome of L. semipalmatus.

Gene	Strand	Position	Overlap/spacer	Size (bp)	Anticodon
*tRNA^Phe^*	H	1-73		73	GAA
s-rRNA	H	74-1046		973	

tRNA^{Val}	H	1047-1118		72	TAC
l-rRNA	H	1119-2716		1598	
tRNA$^{Leu(UUR)}$	H	2717-2790	16	74	TAA
ND1	H	2807-3784		978	
tRNA^{Ile}	H	3785-3854	11	70	GAT
tRNA^{Gln}	L	3866-3936	-1	71	TTG
tRNA^{Met}	H	3936-4004		69	CAT
ND2	H	4005-5043		1039	
tRNA^{Trp}	H	5044-5113	1	70	TCA
tRNA^{Ala}	L	5115-5183	2	69	TGC
tRNA^{Asn}	L	5186-5258	2	73	GTT
tRNA^{Cys}	L	5261-5327	-1	67	GCA
tRNA^{Tyr}	L	5327-5397	1	71	GTA
COX1	H	5399-6949	-9	1551	
tRNA$^{Ser(UCN)}$	L	6941-7014	2	74	TGA
tRNA^{Asp}	H	7017-7085	1	69	GTC
COX2	H	7087-7770	1	684	
tRNA^{Lys}	H	7772-7842	1	71	TTT
ATP8	H	7844-8011	-10	168	
ATP6	H	8002-8685	-1	684	
COX3	H	8685-9468		784	
tRNA^{Gly}	H	9469-9537		69	TCC
ND3	H	9538-9889	4	352	
tRNA^{Arg}	H	9894-9962	1	69	TCG
ND4L	H	9964-10,260	-7	297	
ND4	H	10,254-11,631		1378	
tRNA^{His}	H	11,632-11,700	1	69	GTG
tRNA$^{Ser(AGN)}$	H	11,702-11,765	1	64	GCT
tRNA$^{Leu(CUN)}$	H	11,767-11,837		71	TAG
ND5	H	11,838-13,652	9	1815	
CYTB	H	13,662-14,804	2	1143	
tRNA^{Thr}	H	14,807-14,882	15	76	TGT
tRNA^{Pro}	L	14,898-14,967	19	70	TGG
ND6	L	14,987-15,508	3	522	
tRNA^{Glu}	L	15,512-15,584		73	TTC
D-loop	H	15,585-15,739			

Table S3. Organization of the partial mitochondrial genome of N. arquata.

Gene	Strand	Position	Overlap/spacer	Size (bp)	Anticodon
tRNA^{Phe}	H	1-72		72	GAA
s-rRNA	H	73-1045		973	
tRNA^{Val}	H	1046-1117		72	TAC
l-rRNA	H	1118-2710		1593	
tRNA$^{Leu(UUR)}$	H	2711-2784	12	74	TAA
ND1	H	2797-3774		978	
tRNA^{Ile}	H	3775-3844	12	70	GAT
tRNA^{Gln}	L	3857-3927	-2	71	TTG

$tRNA^{Met}$	H	3926-3995	1	70	CAT
ND2	H	3997-5037	6	1041	
$tRNA^{Trp}$	H	5044-5113	9	70	TCA
$tRNA^{Ala}$	L	5123-5191	2	69	TGC
$tRNA^{Asn}$	L	5194-5267	3	74	GTT
$tRNA^{Cys}$	L	5271-5337	-1	67	GCA
$tRNA^{Tyr}$	L	5337-5407	1	71	GTA
COX1	H	5409-6959	-9	1551	
$tRNA^{Ser(UCN)}$	L	6951-7024	2	74	TGA
$tRNA^{Asp}$	H	7027-7096	1	70	GTC
COX2	H	7098-7781	1	684	
$tRNA^{Lys}$	H	7783-7852	1	70	TTT
ATP8	H	7854-8021	-10	168	
ATP6	H	8012-8695	-1	684	
COX3	H	8695-9478		784	
$tRNA^{Gly}$	H	9479-9547		69	TCC
ND3	H	9548-9898	4	351	
$tRNA^{Arg}$	H	9903-9972		70	TCG
ND4L	H	9973-10,269	-7	297	
ND4	H	10,263-11,639	1	1377	
$tRNA^{His}$	H	11,641-11,709		69	GTG
$tRNA^{Ser(AGN)}$	H	11,711-11,774		64	GCT
$tRNA^{Leu(CUN)}$	H	11,775-11,845		71	TAG
ND5	H	11,846-13,660	10	1815	
CYTB	H	13,671-14,813	2	1143	
$tRNA^{Thr}$	H	14,816-14,887	21	72	TGT
$tRNA^{Pro}$	L	14,909-14,978		70	TGG
ND6	L				
$tRNA^{Glu}$	L				
D-loop	H				

Table S4. Organization of the partial mitochondrial genome of L. limosa.

Gene	Strand	Position	Overlap/spacer	Size (bp)	Anticodon
$tRNA^{Phe}$	H	1-71		71	GAA
s-rRNA	H	72-1043		972	
$tRNA^{Val}$	H	1044-1115		72	TAC
l-rRNA	H	1116-2707		1592	
$tRNA^{Leu(UUR)}$	H	2708-2781	13	74	TAA
ND1	H	2795-3772		978	
$tRNA^{Ile}$	H	3773-3842	9	70	GAT
$tRNA^{Gln}$	L	3852-3922	9	71	TTG
$tRNA^{Met}$	H	3932-4000		69	CAT
ND2	H	4001-5039		1039	
$tRNA^{Trp}$	H	5040-5110	1	71	TCA
$tRNA^{Ala}$	L	5112-5180	12	69	TGC
$tRNA^{Asn}$	L	5193-5265	2	73	GTT
$tRNA^{Cys}$	L	5268-5334	-1	67	GCA

$tRNA^{Tyr}$	L	5334-5404	13	71	GTA
COX1	H	5418-6968	-15	1551	
$tRNA^{Ser(UCN)}$	L	6954-7033	2	80	TGA
$tRNA^{Asp}$	H	7036-7104	1	69	GTC
COX2	H	7106-7789	1	684	
$tRNA^{Lys}$	H	7791-7860	1	70	TTT
ATP8	H	7862-8029	-10	168	
ATP6	H	8020-8703	-1	684	
COX3	H	8703-9486		784	
$tRNA^{Gly}$	H	9487-9555		69	TCC
ND3	H	9556-9907	4	352	
$tRNA^{Arg}$	H	9912-9980	1	69	TCG
ND4L	H	9982-10,278	-7	297	
ND4	H	10,272-11,649		1378	
$tRNA^{His}$	H	11,650-11,718		69	GTG
$tRNA^{Ser(AGN)}$	H	11,720-11,783		64	GCT
$tRNA^{Leu(CUN)}$	H	11,784-11,854		71	TAG
ND5	H	11,855-13,669	11	1814	
CYTB	H	13,681-14,823	1	1143	
$tRNA^{Thr}$	H	14,825-14,898	2	74	TGT
$tRNA^{Pro}$	L	14,901-14,974	14	74	TGG
ND6	L	14,989-15,510	3	522	
$tRNA^{Glu}$	L	15,514-15,587		74	TTC
D-loop	H				

Table S5. The base composition and skew rate of the family Scolopacidae.

Scientific name	Accession	Size (bp)	Proportion of nucleotides (%)					AT skew	GC skew
			T	C	A	G	A+T		
A. hypoleucos	**KM434134**	**16,732**	**25.99**	**29.21**	**31.37**	**13.43**	**57.36**	**0.09**	**-0.37**
A. interpres	AY074885	16,984	24.71	30.72	30.64	13.94	55.34	0.11	-0.38
C. alba	KP742478	16,725	25.09	29.86	31.45	13.60	56.54	0.11	-0.37
C. alpina	KY056596	16,707	25.20	29.83	31.22	13.76	56.41	0.11	-0.37
C. pugnax	KP308149	16,899	26.14	28.77	32.21	12.88	58.35	0.10	-0.38
C. pygmaea	OK552672	17,091	25.40	29.83	30.95	13.83	56.35	0.10	-0.37
C. ruficollis	KY230384	17,047	24.78	29.85	31.88	13.49	56.66	0.13	-0.38
C. subminuta	KX644890	17,668	24.44	30.54	31.61	13.41	56.05	0.13	-0.39
C. tenuirostris	KX230491	16,817	24.91	30.12	31.08	13.90	55.98	0.11	-0.37
C. tenuirostris	KX668223	16,683	24.98	30.01	31.60	13.41	56.58	0.12	-0.38
C. tenuirostris	MF036175	16,906	25.53	29.47	31.62	13.39	57.15	0.11	-0.38
E. pygmeus	KX371106	16,603	24.85	30.02	31.29	13.84	56.14	0.11	-0.37
G.gallinago	KY765409	16,732	25.63	29.65	30.67	14.04	56.31	0.09	-0.36
G. gallinago	MG736926	16,714	25.74	29.44	31.05	13.77	56.78	0.09	-0.36
G. stenura	MW168383	16,860	26.14	28.77	32.21	12.88	58.35	0.10	-0.38
G. stenura	MW168385	16,791	26.33	28.70	32.13	12.83	58.47	0.10	-0.38
L. falcinellus	MK992912	16,765	24.81	30.11	31.50	13.58	56.31	0.12	-0.38
L. lapponica	MW168384	16,642	25.63	29.65	30.67	14.04	56.31	0.09	-0.36
L. lapponica	MW160420	15,555	24.81	30.11	31.50	13.58	56.31	0.12	-0.38

N. madagascariensis	KY128485	16,804	25.89	29.36	30.94	13.80	56.84	0.09	-0.36
N. madagascariensis	MK460251	16,682	24.93	29.82	31.81	13.44	56.74	0.12	-0.38
N. minutus	MN956840	16,902	25.36	29.69	31.31	13.64	56.67	0.11	-0.37
N. phaeopus	KY434065	16,709	25.40	29.83	30.95	13.83	56.35	0.10	-0.37
N. tenuirostris	MK341548	16,732	24.64	30.51	30.92	13.93	55.57	0.11	-0.37
P. lobatus	MW160419	16,678	25.74	29.44	31.05	13.77	56.78	0.09	-0.36
P. parvirostris	MZ157405	16,919	26.17	28.59	32.53	12.71	58.70	0.11	-0.38
S. rusticola	MW865755	16,814	25.02	29.85	31.79	13.34	56.81	0.12	-0.38
T. erythropus	KY888681	18,153	24.98	30.01	31.60	13.41	56.58	0.12	-0.38
T. glareola	MT188757	16,695	25.65	29.26	31.67	13.41	57.33	0.10	-0.37
T. guttifer	MK341549	16,773	25.52	29.71	30.79	13.99	56.30	0.09	-0.36
T. nebularia	MW930394	17,117	24.93	29.82	31.81	13.44	56.74	0.12	-0.38
T. nebularia	MK108195	16,705	25.40	29.72	30.87	14.02	56.26	0.10	-0.36
T. ochropus	MT880247	15,590	25.53	29.47	31.62	13.39	57.15	0.11	-0.38
T. semipalmata inornata	MK905885	16,835	25.24	29.89	31.24	13.64	56.47	0.11	-0.37
T. stagnatilis	MG883743	16,689	25.15	29.92	31.59	13.34	56.74	0.11	-0.38
T. totanus	MT572847	16,799	25.46	29.52	31.52	13.50	56.98	0.11	-0.37
X. cinereus	MK922124	16,818	24.86	30.17	30.71	14.27	55.56	0.11	-0.36
Average		16,793	25.32	29.71	31.39	13.58	56.71	0.11	-0.37

Table S6. The base composition and skew rate of PCGs.

Species	Gene	Size(bp)	Proportion of nucleotides (%)					AT skew	GC skew
			T	C	A	G	A+T		
**A. hypoleucos**	*ND1*	978	31.08	28.53	28.32	12.07	59.41	-0.05	-0.41
	ND2	1041	27.33	30.13	32.72	9.82	60.06	0.09	-0.50
	COX1	1551	28.63	28.11	27.21	16.05	55.83	-0.03	-0.27
	COX2	684	26.75	28.36	30.70	14.18	57.46	0.07	-0.33
	ATP8	168	22.62	36.90	35.71	4.76	58.33	0.22	-0.77
	ATP6	684	27.34	34.06	28.51	10.09	55.85	0.02	-0.54
	COX3	784	27.55	28.32	28.70	15.43	56.25	0.02	-0.29
	ND3	352	29.34	29.34	29.63	11.68	58.97	0.00	-0.43
	ND4L	297	26.94	30.98	29.63	12.46	56.57	0.05	-0.43
	ND4	1378	25.54	33.09	32.15	9.22	57.69	0.11	-0.56
	ND5	1815	24.46	31.79	32.89	10.85	57.36	0.15	-0.49
	CYTB	1143	27.38	31.93	29.05	11.64	56.43	0.03	-0.47
	ND6	522	42.72	9.39	12.64	35.25	55.36	-0.54	0.58
**L. semipalmatus**	*ND1*	978	28.32	32.00	26.99	12.68	55.32	-0.02	-0.43
	ND2	1041	24.16	33.97	31.95	9.91	56.11	0.14	-0.50
	COX1	1551	27.60	28.82	27.92	15.67	55.51	0.01	-0.30
	COX2	684	25.73	28.95	31.43	13.89	57.16	0.10	-0.35
	ATP8	168	25.00	35.12	34.52	5.36	59.52	0.16	-0.74
	ATP6	684	23.83	37.43	28.36	10.38	52.19	0.09	-0.57
	COX3	784	26.66	29.72	27.55	16.07	54.21	0.02	-0.30
	ND3	352	27.35	31.34	28.77	12.54	56.13	0.03	-0.43
	ND4L	297	25.59	32.66	27.95	13.80	53.54	0.04	-0.41

	ND4	1378	25.11	33.74	31.13	10.01	56.24	0.11	-0.54
	ND5	1815	24.63	31.40	32.95	11.02	57.58	0.14	-0.48
	CYTB	1143	26.77	32.46	29.05	11.72	55.82	0.04	-0.47
	ND6	522	42.53	8.43	12.84	36.21	55.36	-0.54	0.62
N. arquata	*ND1*	978	28.94	30.78	27.40	12.88	56.34	-0.03	-0.41
	ND2	1039	25.36	32.47	32.76	9.41	58.12	0.13	-0.55
	COX1	1551	25.98	30.63	26.95	16.44	52.93	0.02	-0.30
	COX2	684	25.44	29.39	30.12	15.06	55.56	0.08	-0.32
	ATP8	168	27.98	32.74	33.93	5.36	61.90	0.10	-0.72
	ATP6	684	26.02	35.23	28.22	10.53	54.24	0.04	-0.54
	COX3	784	27.93	28.32	27.93	15.82	55.87	0.00	-0.28
	ND3	351	27.07	31.91	28.49	12.54	55.56	0.03	-0.44
	ND4L	297	28.28	28.96	27.61	15.15	55.89	-0.01	-0.31
	ND4	1377	25.13	33.70	30.36	10.82	55.48	0.09	-0.51
	ND5	1815	25.40	31.40	32.34	10.85	57.74	0.12	-0.49
	CYTB	1143	26.33	32.55	28.78	12.34	55.12	0.04	-0.45
L. limosa	*ND1*	978	27.81	31.60	26.89	13.70	54.70	-0.02	-0.40
	ND2	1041	25.99	32.05	31.67	10.30	57.65	0.10	-0.51
	COX1	1551	27.21	29.34	27.40	16.05	54.61	0.00	-0.29
	COX2	684	25.73	29.97	29.53	14.77	55.26	0.07	-0.34
	ATP8	168	23.81	36.90	35.71	3.57	59.52	0.20	-0.82
	ATP6	684	25.88	35.67	27.78	10.67	53.65	0.04	-0.54
	COX3	784	27.81	28.06	29.08	15.05	56.89	0.02	-0.30
	ND3	352	25.64	33.05	29.91	11.40	55.56	0.08	-0.49
	ND4L	297	26.94	30.64	26.94	15.49	53.87	0.00	-0.33
	ND4	1378	25.25	33.89	30.48	10.38	55.73	0.09	-0.53
	ND5	1814	23.86	32.34	32.51	11.29	56.36	0.15	-0.48
	CYTB	1143	25.13	34.15	28.28	12.43	53.42	0.06	-0.47
	ND6	522	41.38	10.54	13.79	34.29	55.17	-0.50	0.53

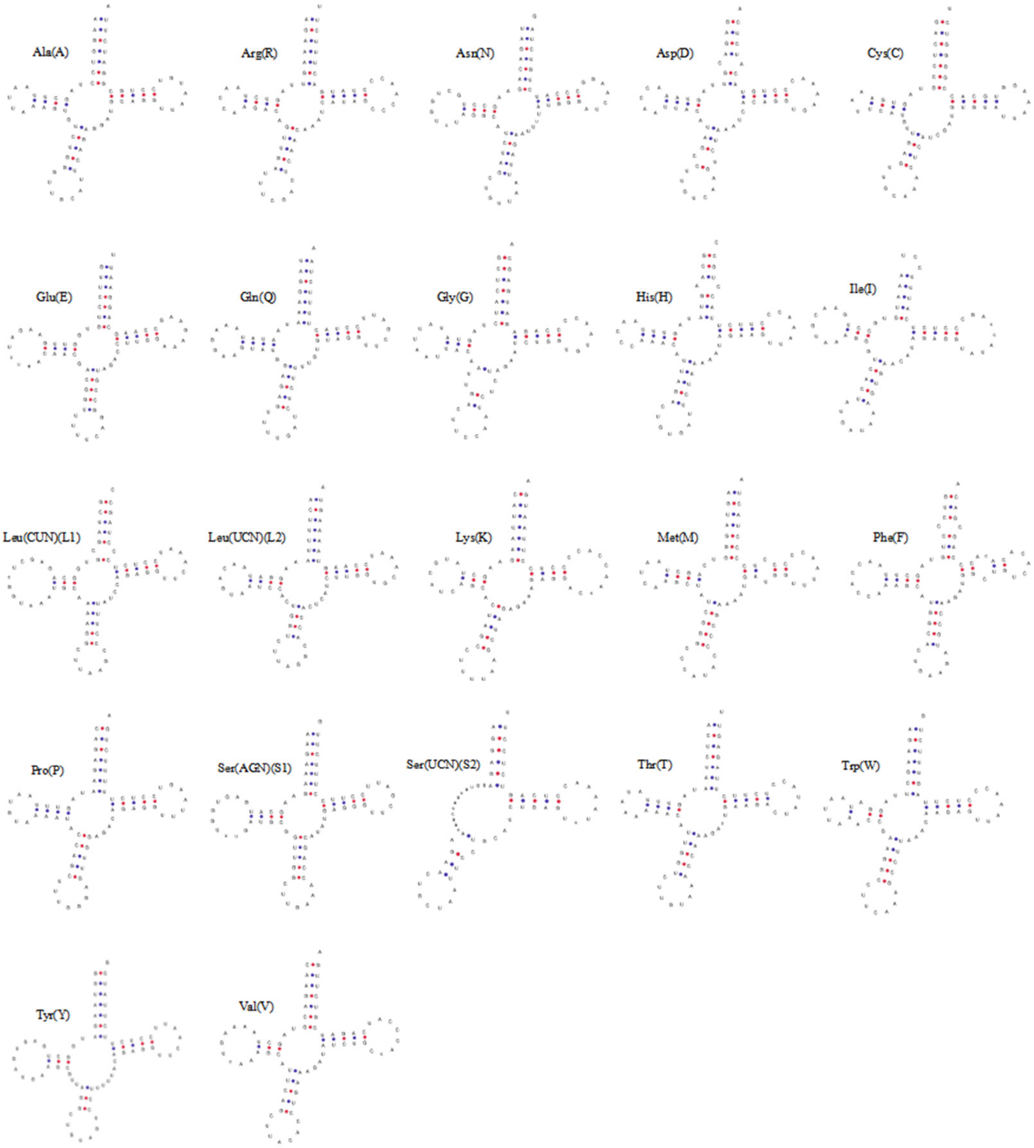

Figure S1. Secondary structures of the 22 tRNA genes of *A. hypoleucos*.

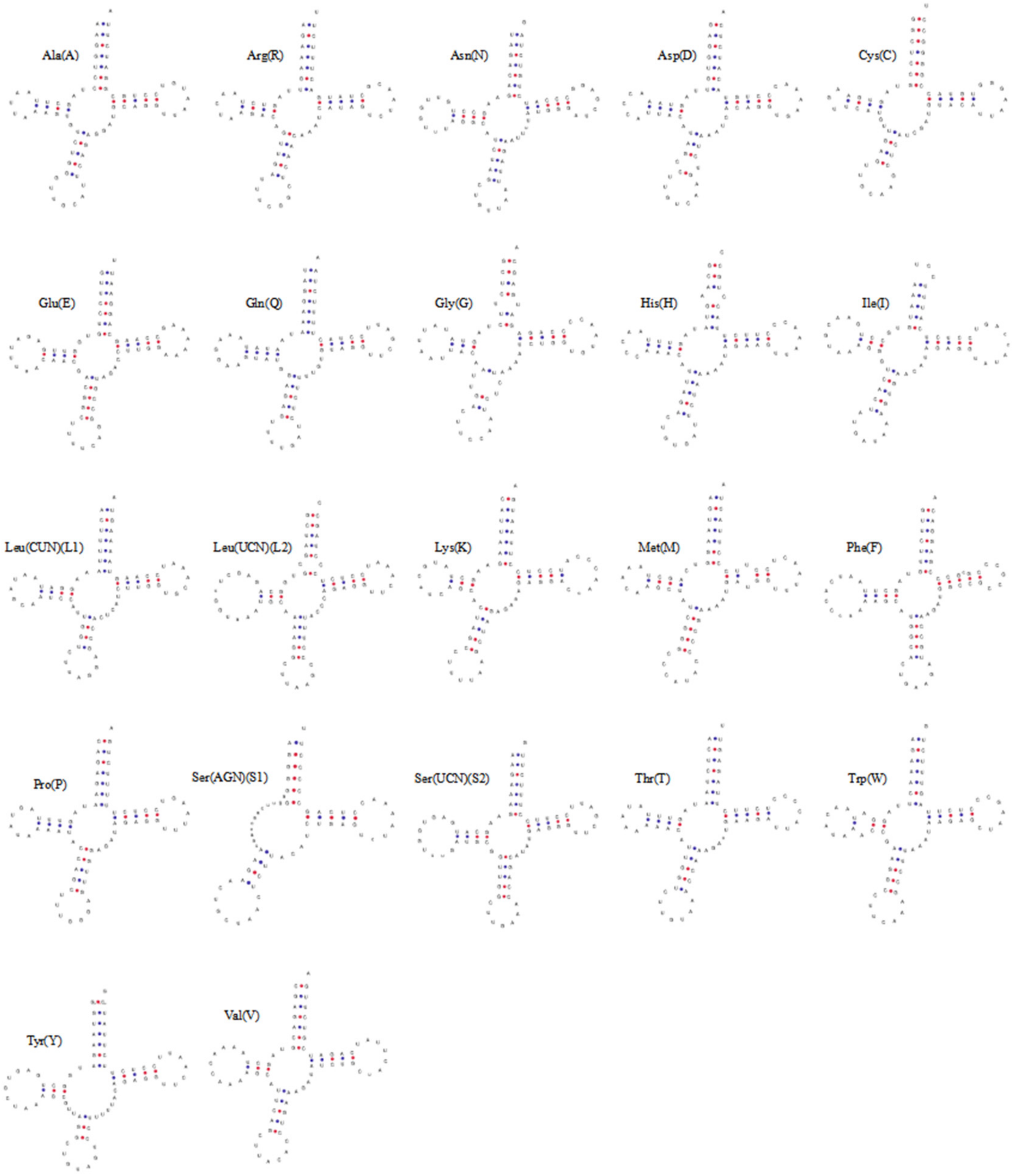

Figure S2. Secondary structures of the 22 tRNA genes of *L. semipalmatus*.

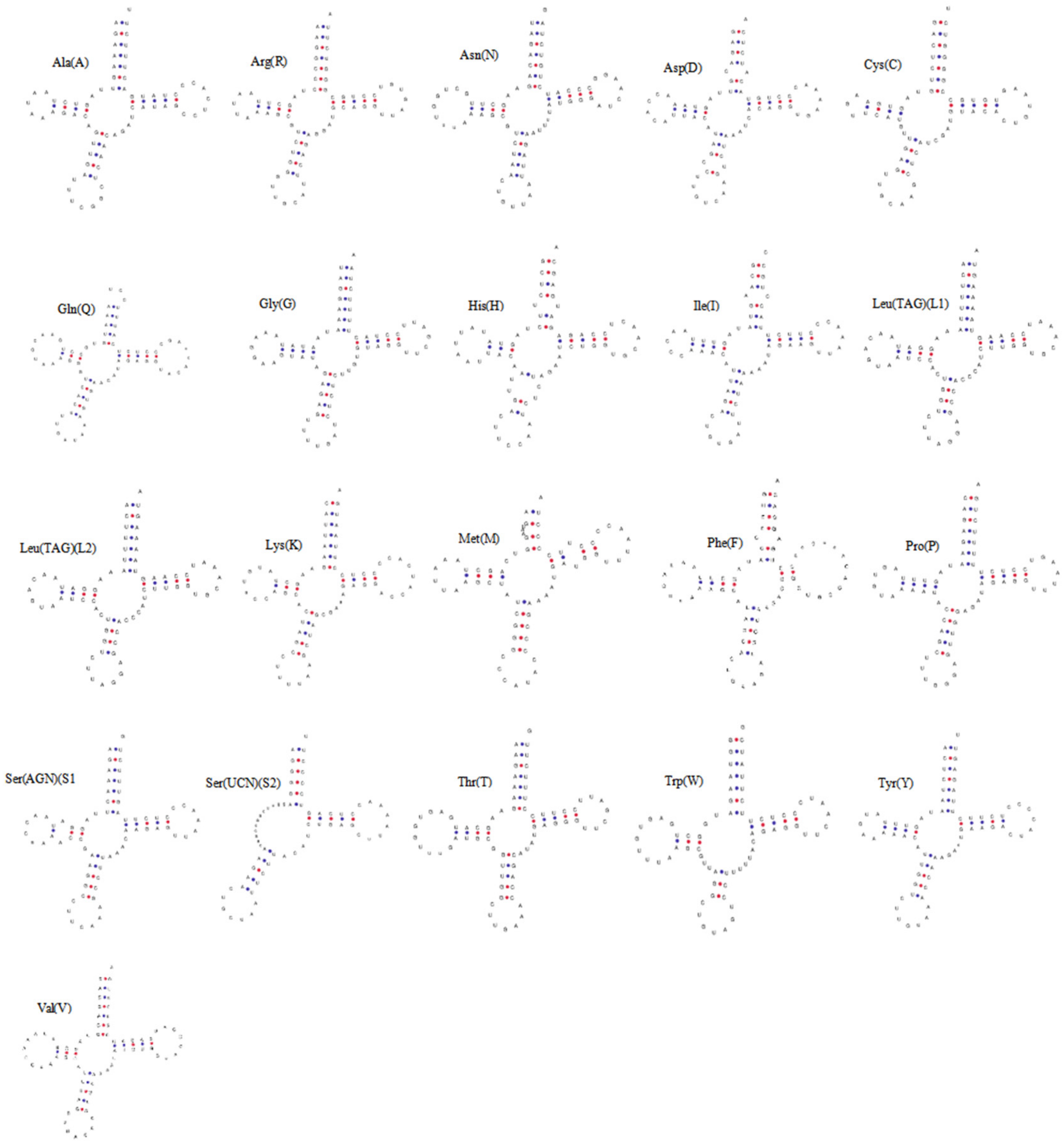

Figure S3. Secondary structures of the 21 tRNA genes of N. arquata.

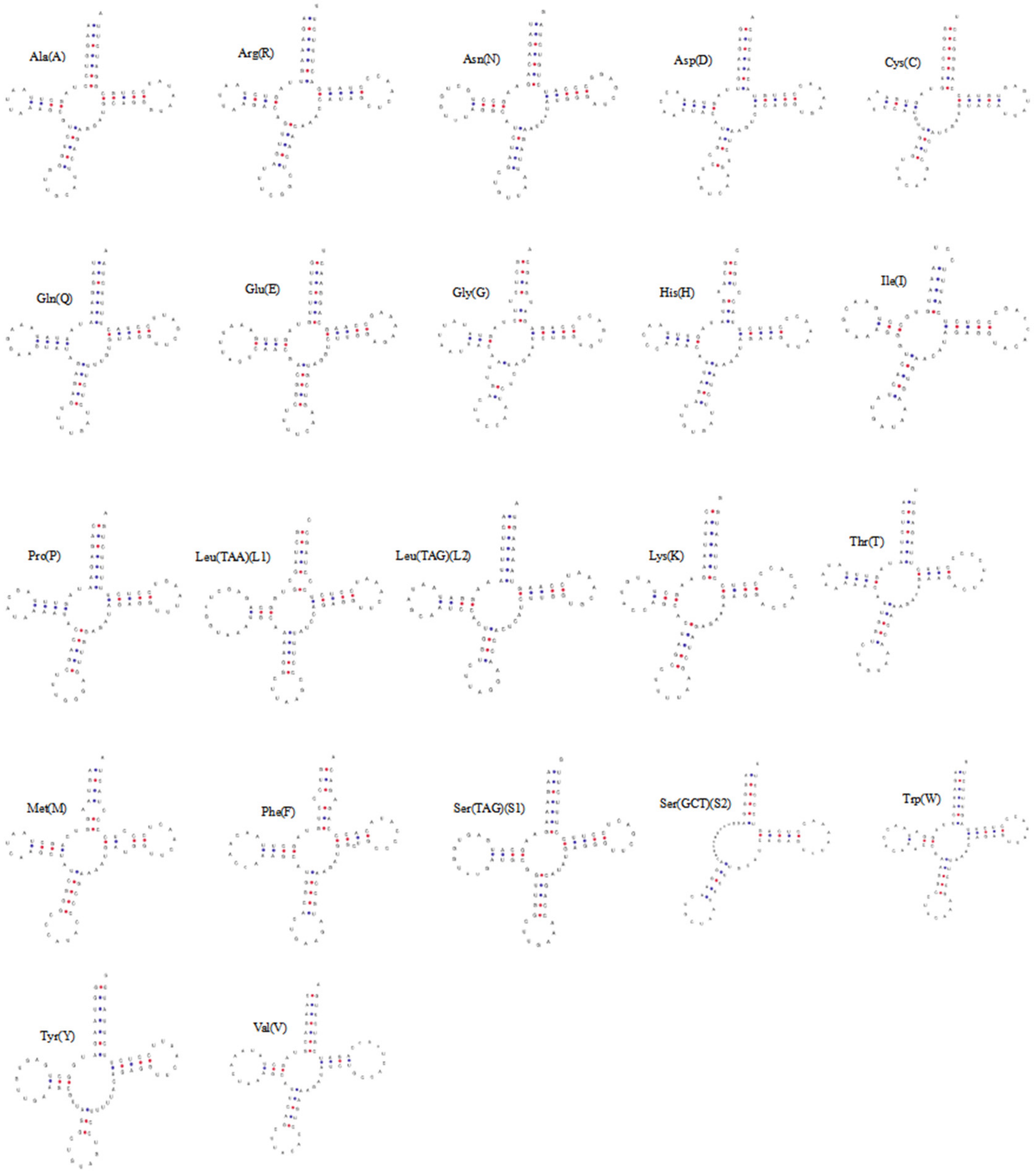

Figure S4. Secondary structures of the 22 tRNA genes of L. limosa.

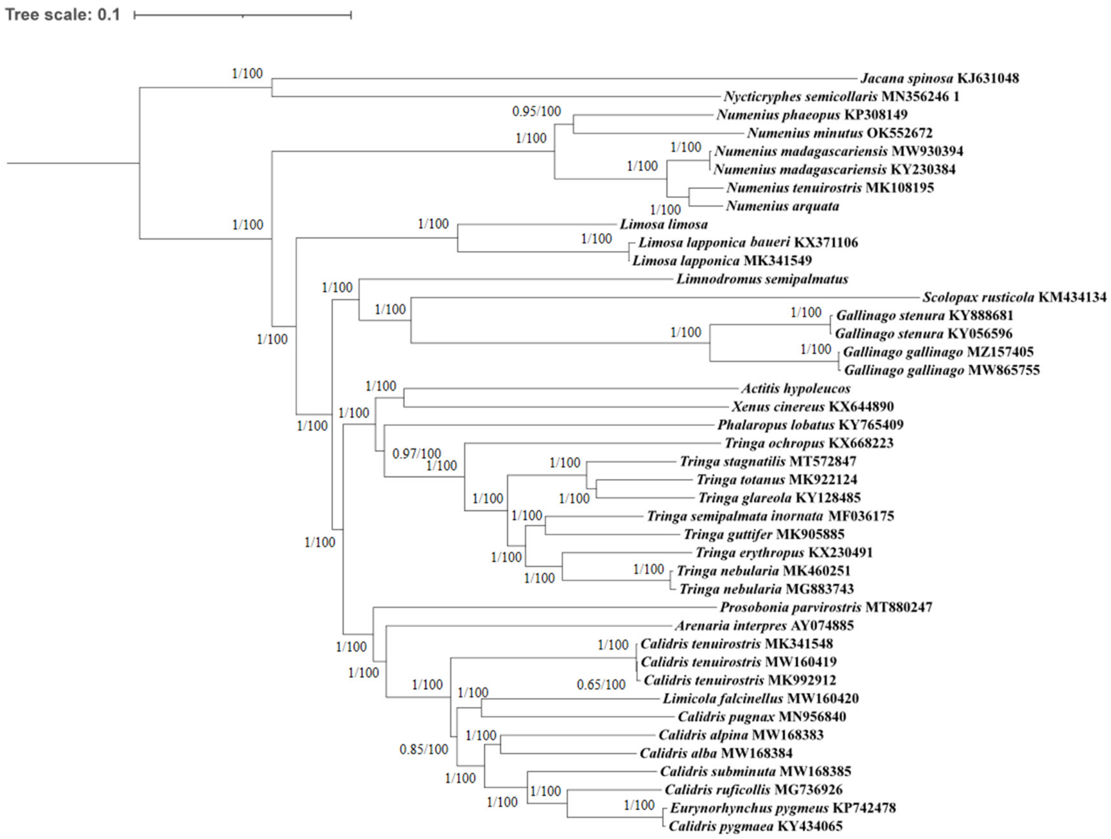

Figure S5. Phylogenetic relationships among Scolopacidae species based on mitochondrial genes (concatenated 12 protein-coding genes, 12S and 16S rRNA), using Bayesian inference (BI) and Maximum likelihood (ML). Numbers at nodes represent the Bayesian percent posterior probabilities and Maximum likelihood bootstrap values, separated by '/'.
